# Integrated slice-specific dynamic shimming diffusion weighted imaging (DWI) for rectal Cancer detection and characterization

**DOI:** 10.1186/s40644-021-00403-9

**Published:** 2021-04-07

**Authors:** Jianxing Qiu, Jing Liu, Zhongxu Bi, Xiaowei Sun, Xin Wang, Junling Zhang, Chengwen Liu, Jinxia Zhu, Naishan Qin

**Affiliations:** 1 Department of Radiology, Peking University First Hospital, 8 XiShiKu Avenue, XiCheng District, Beijing, 100034 China; 2 Department of Gastrointestinal Surgery, Peking University First Hospital, Beijing, China; 3 MR Collaboration, Siemens Healthcare, Ltd., Beijing, China

**Keywords:** Rectal Cancer, Integrated slice-specific dynamic shimming (iShim); diffusion weighted imaging (DWI), Imaging quality, Tumor differentiation

## Abstract

**Purpose:**

To compare integrated slice-specific dynamic shimming (iShim) diffusion weighted imaging (DWI) and single-shot echo-planar imaging (SS-EPI) DWI in image quality and pathological characterization of rectal cancer.

**Materials and methods:**

A total of 193 consecutive rectal tumor patients were enrolled for retrospective analysis. Among them, 101 patients underwent iShim-DWI (b = 0, 800, and 1600 s/mm^2^) and 92 patients underwent SS-EPI-DWI (b = 0, and 1000 s/mm^2^). Qualitative analyses of both DWI techniques was performed by two independent readers; including adequate fat suppression, the presence of artifacts and image quality. Quantitative analysis was performed by calculating standard deviation (SD) of the gluteus maximus, signal intensity (SI) of lesion and residual normal rectal wall, apparent diffusion coefficient (ADC) values (generated by b values of 0, 800 and 1600 s/mm^2^ for iShim-DWI, and by b values of 0 and 1000 s/mm^2^ for SS-EPI-DWI) and image quality parameters, such as signal-to-noise ratio (SNR) and contrast-to-noise ratio (CNR) of primary rectal tumor. For the primary rectal cancer, two pathological groups were divided according to pathological results: Group 1 (well-differentiated) and Group 2 (poorly differentiated). Statistical analyses were performed with *p* < 0.05 as significant difference.

**Results:**

Compared with SS-EPI-DWI, significantly higher scores of image quality were obtained in iShim-DWI cases (*P* < 0.001). The SD_background_ was significantly reduced on b = 1600 s/mm^2^ images and ADC maps of iShim-DWI. Both SNR and CNR of b = 800 s/mm^2^ and b = 1600 s/mm^2^ images in iShim-DWI were higher than those of b = 1000 s/mm^2^ images in SS-EPI-DWI. In primary rectal cancer of iShim-DWI cohort, SI_lesion_ was significantly higher than SI_rectum_ in both b = 800 and 1600 s/mm^2^ images. ADC values were significantly lower in Group 2 (0.732 ± 0.08) × 10^− 3^ mm^2^/s) than those in Group 1 ((0.912 ± 0.21) × 10^− 3^ mm^2^/s). ROC analyses showed significance of ADC values and SI_lesion_ between the two groups.

**Conclusion:**

iShim-DWI with b values of 0, 800 and 1600 s/mm^2^ is a promising technique of high image quality in rectal tumor imaging, and has potential ability to differentiate rectal cancer from normal wall and predicting pathological characterization.

**Supplementary Information:**

The online version contains supplementary material available at 10.1186/s40644-021-00403-9.

## Key results

1. iShim-DWI improves images quality of rectal cancer compared with SS-EPI-DWI.
2. ADC Values generated by b values of 0, 800 and 1600 s/mm^2^ in iShim-DWI can help differentiate high-grade cancer from low-grade cancer.

## Introduction

Diffusion-weighted imaging (DWI) is increasingly used for detection and characterization of rectal cancer in clinical practice [1–5]. Single-shot echo-planar imaging (SS-EPI) DWI has been routinely used as the main DWI sequence. However, SS-EPI-DWI has limited spatial resolution and is sensitive to motion and magnetic field inhomogeneity [6, 7]. In addition, susceptibility artifacts from gas in rectum may also cause image distortion. Advancement in DWI technology makes it possible to perform DWI with ultra-high b value or multiple b-values to obtain more information for lesion detection and characterization [8–12]. The most widely used high b-values for DWI in rectal cancer are 800 and 1000 s/mm^2^ in routine work [11, 13–15]. Lower b-values are always associated with a risk of ‘T2-shine-through’ effects. DWI with ultra-high b-values (above 1000 s/mm2) might allow for a better visualization of tumors due to a highly effective suppression of background signal [9–12]. A previous study using DWI with b value of 2000 s/mm ^2^ in rectal cancer imaging suggested that signal intensity (SI) helps assess the primary rectal tumor and its response to chemo-radiation therapy (CRT) [10]. However, the resolution of images with ultra-high b value is limited for clinical diagnosis. A solution is to combine high and ultra-high b values to balance spatial resolution and functional information. Chen et al., [11] investigated different b-value combination and suggested the apparent diffusion coefficient (ADC) value generated by combining high and ultra-high b values shows improvement in rectal cancer characterization in 32 patients.

Recently, integrated slice-specific dynamic shimming (iShim) DWI has been reported to improve image quality and lesion detection in bladder, prostate, breast, and thyroid compared with SS-EPI-DWI [16–20]. In this study, we investigate the performance of iShim-DWI in detection and characterization of rectal cancer, in comparison with SS-EPI-DWI technology.

## Material and methods

### Ethics statement

This study was approved by institutional review board. The need for written informed consent was waived by the institutional review board due to the retrospective design of the study.

### Patients enrollment

A total of 204 consecutive patients with suspected rectal cancer by surgeon or histologically proven rectal cancer were referred for a multiparametric MR examination from June 2018 to June 2020. Among them, 2 patients received both SS-EPI-DWI and iShim-DWI to testify the feasibility of iShim-DWI.

Among the rest 202 patients, 107 consecutive cases were imaged with iShim-DWI, and 95 consecutive cases with SS-EPI-DWI. Consecutive patients were selected for each cohort. The inclusion criteria for rectal cancer were as follows: proven rectal tumor by either biopsy or surgery (including both primary tumor and after treatment). The exclusion criteria were as follows: non-rectal tumor confirmed by endoscopy at the first time.

**Fig. 1:**
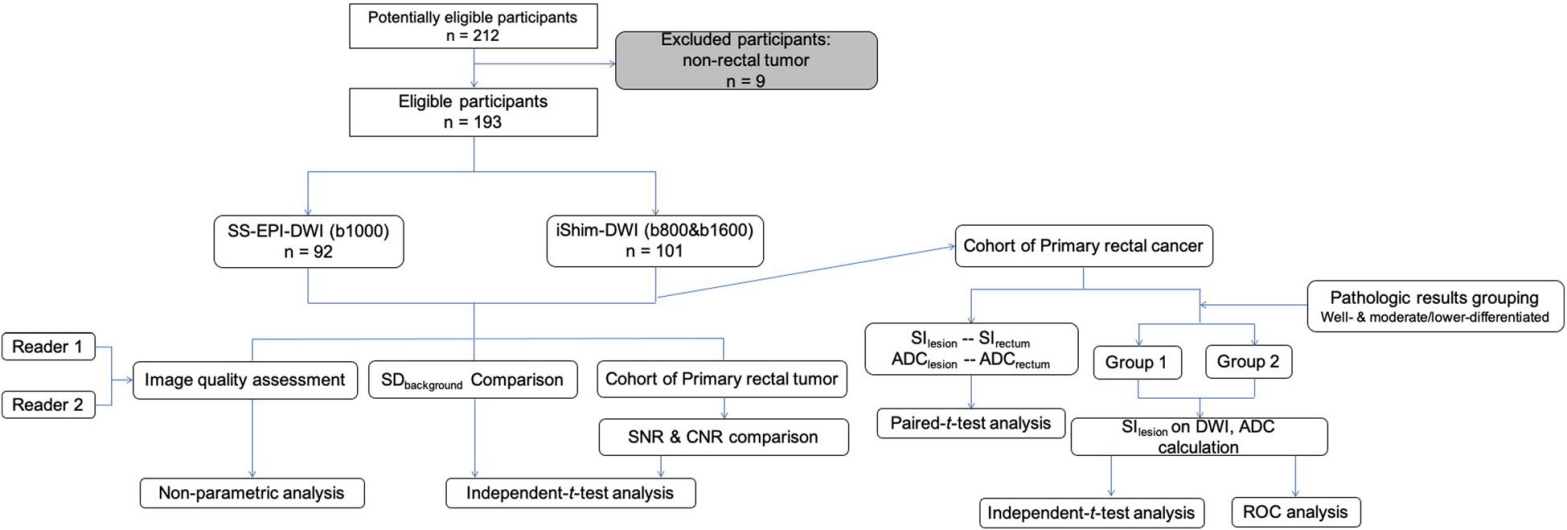
Work Flowchart

Among the 107 iShim-DWI cases, 6 cases were excluded due to ulcerative colitis (3), rectal inflammation with unknown reason (2), and rectum invaded by fallopian tube carcinoma (1). Among the SS-EPI-DWI cases, 3 patients were excluded due to epidermoid cyst (1), intussusception (1), and radiation enteritis from cervical carcinoma (1) (Fig. 1).

### MRI acquisition

All MRI examinations were performed on a MAGNETOM Aera 1.5 T MRI scanner (Siemens Healthcare, Erlangen, Germany) with an 18-channel body coil and a 32-channel spine coil. No special bowel preparation was applied. The rectal cancer examination protocols included T2-weighted Turbo Spin Echo (T2W TSE) images in axial, sagittal, coronal orientation, and DWI using a prototype iShim-DWI (Siemens Healthcare, Erlangen, Germany) or standard SS-EPI-DWI. ADC maps were calculated inline for each DWI sequence with a monoexponentially fit based on the 3 or 2 measured b-values. Then ten dynamic enhanced T1W images were collected after administering body weight-adapted intravenous gadopentetate dimeglumine (0.1 mmol/kg BW, Dotarem; Guerbet, Paris, France) at a rate of 2 mL/s. Protocol parameters for these sequences were summarized in Supplement 1.

### Qualitative assessment

Images were interpreted using the Picture Archiving and Communication Systems (PACS) workstations. Two experienced radiologists (Reader 1 with 10 years and Reader 2 with 20 years of experience in abdominal MRI) who were unaware of patients’ clinical data assessed DWI images independently, including both iShim DWI and SS-EPI-DWI, and rated the image quality of DWI images (b1000 for SS-EPI-DWI, both b = 800 and 1600 s/mm^2^ for iShim-DWI), ADC maps for each patient according to a Likert-type scale:

1. Adequate fat suppression for DW images:

- Score 1: failure in suppression;
- Score 2: regional fat-water failures but still interpretable;
- Score 3: minimal failures in image periphery;
- Score 4: perfect fat-water separation

2) Presence of artifact or artifacts for both DW images and ADC maps:

- Score 1: nondiagnostic;
- Score 2: artifacts but diagnostic;
- Scroe 3: no artifact.

3) Image quality for both DW images and ADC maps:

- Score 1: nondiagnostic image quality with serious artifacts, image distortion, or poor signal intensity.
- Score 2: poor diagnostic image quality and diagnostic confidence of the readers, with serious artifacts, image distortion pronounced, or relatively poor signal intensity.
- Score 3: moderate image quality and confidence of the readers: with some artifacts, moderate image distortion.
- Score 4: good diagnostic image quality and confidence of the readers: with few artifacts, slight image distortion, anatomic structures (e.g. rectal wall) well delineated.
- Score 5: excellent image quality and strong confidence of the readers in the diagnosis: with almost no artifacts or imaging distortion.

The 2 readers assessed the images separately in a blinded, randomized fashion. The discrepancies were resolved in a consensus reading. The results of the consensus reading were used for statistical analyses.

### Quantitative assessment

Reader 1 calculated the SI on DW images and ADC values on ADC maps in all the patients using PACS.

For ADC maps:

1. Two region of interests (ROIs) of no less than 20 mm^2^ were placed on different section of the lesion. The ROIs were manually drawn tracing along the margin of the lesion on ADC map, and to maximumly include the whole solid part of lesion with avoiding necrosis and hemorrhage. The average value caculated by the two ROIs were regarded as the mean ADC_lesion_.
2. Two ROIs of nearly 10 mm^2^ were placed on different part of the residual normal rectum. The average values caculated by the two were regarded as the mean ADC_rectum_.
3. One ROI of 100 mm^2^ was placed on the right gluteus maximus. The standard deviation (SD) was regarded as the background noise: SD_background-ADC_ for ADC map.

For DW Images:

1. Two ROIs of no less than 20 mm^2^ were placed in the lesion (for SS-EPI-DWI, drawn on images of b = 1000 s/mm^2^, and for iShim-DWI, drawn on both images of b = 800 and 1600 s/mm^2^) with the same location of ADC maps. The average values caculated by the two ROIs were regarded as SI_lesion_ separetely.
2. Two ROIs of nearly 10 mm^2^ were placed on different part of the residual normal rectum with the same location in ADC maps. The average values caculated by the two ROIs were regarded as SI_rectum_.
3. One ROI of 100 mm^2^ was placed on the right gluteus maximus with the same location in ADC maps. The SD was regarded as the background noise: SD_background-DWI_ for DW images.

Signal-noise ratio (SNR) and contrast-to-noise ratio (CNR), defined below, were calculated for each DW images.

$$ \mathrm{SNR}={\mathrm{SI}}_{\mathrm{lesion}}/{\mathrm{SD}}_{\mathrm{background}-\mathrm{DWI}} $$

$$ \mathrm{CNR}=\left({\mathrm{SI}}_{\mathrm{lesion}}-{\mathrm{SI}}_{\mathrm{rectum}}\right)/{\mathrm{SD}}_{\mathrm{background}-\mathrm{DWI}} $$

### Pathological grouping for primary rectal cancer

For the primary rectal cancer, 2 pathological groups were be divided according to their surgical pathological results in cohorts of iShim-DWI and SS-EPI-DWI, respectively:

Group 1 (rectal cancer with relatively well differentiation): patients with carcinoma in situ and rectal cancer of Grade 1 (high differentiated in pathology).

Group 2 (rectal cancer with relatively poor differentiation): patients with Grade 2 (moderate differentiated in pathology) and Grade 3 (poor differentiated in pathology).

### Statistical analysis

SPSS software (version 24.0; SPSS, Chicago, Ill) was used for the statistical analysis. Firstly, nonparametric analysis (Mann-Whitney U tests) were applied to assess fat suppression, artifacts and image quality and to compare iShim- and SS-EPI-DWI, ADC maps. Secondly, Independent-*t*-test analysis was applied to assess quantitative SD_background_ (both DWI and ADC maps), SNR and CNR of iShim- and SS-EPI-DWI.

For primary rectal tumor, paired-*t*-test analysis was applied between lesions and normal rectums for both (SI_lesion_-SI_rectum_) and (ADC_lesion_-ADC_rectum_) to explore if SI or ADC value of iShim could distinguish lesions from normal rectum. The analysis was conducted in cohort of iShim-DWI and SS-EPI-DWI, respectively.

For primary rectal cancer, independent-*t*-test analysis was applied for SI_lesion_ of DWI, ADC_lesion_ between two pathologic groups in cohort of iShim-DWI and SS-EPI-DWI, respectively. Finally, receiver operator curve (ROC) analysis was performed in SI_lesion_ and ADC_lesion_ between the two groups to explore if DWI (both iShim- and SS-EPI-DWI) could help differentiate them. *P* < 0.05 was considered indicative of a significant difference.

## Results

### Results of feasibility experiment

In the 2 patients performed with both iShim- and SS-EPI-DWI, higher scores regarding adequate fat suppression and image quality were obtained in iShim-DWI (both images of b800 and b1600). Scores of 3.5, 4 and 4 were obtained in fat supprestion for images of b = 1000, 800 and 1600 s/mm^2^, respectively. Scores of 4.5, 5 and 5 were obtained in image quality for images of b = 1000, 800 and 1600 s/mm^2^, respectively. In addition, average SNR of lesions were 21.5, 38.4 and 30.2 for images of b = 1000, 800 and 1600 s/mm^2^, respectively. Average CNR of lesions were 10.6, 16.8 and 15.9 for images of b = 1000, 800 and 1600 s/mm^2^, respectively.

For ADC maps, higher scores regarding image quality were also obtained in iShim-DWI compared with SS-EPI-DWI. Scores of 4.5, 5 and 5 were obtained in image quality for images of b = 1000, 800 and 1600 s/mm^2^, respectively.

### Patients and clinical information

Among 101 patients underwent iShim-DWI, 63 were males and 38 were females, with the mean age of 62.1 ± 11.4 years (32 to 83 years); 73 patients were primary rectal tumor while 28 patients were post-surgery or CRT.

Among the 92 patients underwent SS-EPI-DWI, 64 were males and 28 were females, with the mean age of 60.9 ± 12.6 years (24 to 93 years); 65 patients were primary rectal tumor while the rest 27 patients were post-surgery or CRT.

### Pathological results for primary rectal tumor of iShim-DWI

Pathological results were obtained for all the primary rectal tumor in both cohorts of iShim-DWI and SS-EPI-DWI.

In patients of iShim-DWI, 7 patients were confirmed as non-adenocarcinoma (1 for solitary fibroma, 2 for stromal tumor, 2 for benign adenoma, and 2 for neuroendocrine tumor). The rest 66 patients were confirmed as primary rectal adenocarcinoma (Carcinoma in situ (*n* = 8), Grade 1 (*n* = 3), Grade 2 (*n* = 47), Grade 3 (*n* = 8)) for the pathological grouping. Among them, 11 patients were included in Group 1 and 55 patients were included in Group 2.

In patients of SS-EPI-DWI, 2 patients were confirmed as non-adenocarcinoma (1 for malignant melanoma, 1 for neuroendocrine tumor), 3 patients were confirmed as mucinous adenocarcinoma. The rest 60 patients were confirmed as primary rectal cancer (Carcinoma in situ (*n* = 1), Grade 1 (*n* = 4), Grade 2 (*n* = 52), Grade 3 (*n* = 3)) for the pathological grouping. Among them, 5 patients were included in Group 1 and 55 patients were included in Group 2.

### Qualitative assessment

For DW images comparison, significantly higher scores regarding adequate fat suppression and image quality were obtained in patients using iShim-DWI (both images of b = 800 and 1600 s/mm^2^) in all the cohorts (total cohort, cohort of primary rectal tumor and cohort of after treatment) by Mann-Whitney U tests. No significant differences were obtained for the comparison of artifacts between iShim- and SS-EPI-DWI (Table 1).

**Table 1:** Results of Qualitative Image quality comparison between iShim- and SS-EPI-DWI

			Fat suppression			Artifacts			Imaging quality		
Cohort			Scores	*Z*	*P*	Scores	*Z*	*P*	Scores	*Z*	*P*
Total	DWI	b1000	3.25 ± 0.51	–		2.87 ± 0.45	–		4.35 ± 0.53	–	
		b800	3.92 ± 0.27	−9.078	< 0.001*	2.94 ± 0.24	−0.804	0.422	4.82 ± 0.38	−6.439	< 0.001*
		b1600	3.94 ± 0.24	−9.402	< 0.001*	2.92 ± 0.27	−0.281	0.779	4.88 ± 0.33	−7.381	< 0.001*
	ADC Map	SS-EPI	–			2.91 ± 0.28	−1.034	0.301	4.3 ± 0.46	−6.946	< 0.001*
		iShim				2.95 ± 0.21			4.8 ± 0.4		
Primary rectal tumor	DWI	b1000	3.34 ± 0.51	–		2.89 ± 0.4	–		4.42 ± 0.56	–	
		b800	3.97 ± 0.16	−7.75	< 0.001*	2.96 ± 0.2	−9.27	0.354	4.9 ± 0.3	−5.796	< 0.001*
		b1600	3.97 ± 0.16	−7.75	< 0.001*	2.93 ± 0.25	−0.237	0.812	4.93 ± 0.25	−6.214	< 0.001*
	ADC Map	SS-EPI	–			2.92 ± 0.27	−0.896	0.37	4.34 ± 0.48	−6.49	< 0.001*
		iShim				2.96 ± 0.2			4.88 ± 0.33		
After treatment	DWI	b1000	3.04 ± 0.43	–		2.81 ± 0.56	–		4.19 ± 0.4	–	
		b800	3.79 ± 0.42	−4.994	< 0.001*	2.89 ± 0.31	−0.14	0.889	4.61 ± 0.5	−3.164	0.002*
		b1600	3.86 ± 0.36	−5.465	< 0.001*	2.89 ± 0.31	−0.14	0.889	4.75 ± 0.44	−4.156	< 0.001*
	ADC Map	SS-EPI	–			2.89 ± 0.32	−0.507	0.612	4.22 ± 0.42	−2.867	0.004*
		iShim				2.93 ± 0.26			4.61 ± 0.5		

*P* with * suggested statistical significance. All the t and *P* values are the comparison between b = 800 s/mm^2^ of iShim-DWI and b = 1000 s/mm^2^ of SS-EPI-DWI, b = 1600 s/mm^2^ of iShim-DWI and b = 1000 s/mm^2^ of SS-EPI-DWI for each DWI item

For ADC maps, significantly higher scores regarding image quality were obtained in patients using iShim-DWI in all the cohorts. And significantly higher score regarding artifacts was obtained in the cohort of primary rectal tumor using iShim-DWI by Mann-Whitney U tests (Table 1).

### Quantitative assessment

SD_background_ showed significantly decreasing in b = 1600 s/mm^2^ images and ADC maps of iShim-DWI in all the cohorts, compared with SS-EPI-DWI by paired-t-test analyses (Table 2).

**Table 2:** Results of Quantitative Image quality comparison between iShim- and SS-EPI-DWI

			SD_**background**_			SNR			CNR		
Cohort			Values	*t*	*P*	Ratio	*t*	*P*	Ratio	*t*	*P*
Total	DWI	b1000	5.99 ± 1.59	–		–			–		
		b800	6.34 ± 2.49	1.144	0.254						
		b1600	4.53 ± 1.51	−6.54	< 0.001*						
	ADC Map	SS-EPI	174.68 ± 116.46	−7.174	< 0.001*						
		iShim	88.3 ± 31.43								
Primary rectal tumor	DWI	b1000	5.96 ± 1.56	–		22.57 ± 8.66	–		10.81 ± 5.83	–	
		b800	6.11 ± 2.44	0.436	0.063	41.26 ± 17.02	8.157	< 0.001*	21.53 ± 11.46	6.948	< 0.001*
		b1600	4.49 ± 1.54	−5.542	< 0.001*	35.35 ± 14.76	6.208	< 0.001*	22.59 ± 11.95	7.39	< 0.001*
	ADC Map	SS-EPI	179.71 ± 134.98	−5.46	< 0.001*	–			–		
		iShim	91.2 ± 29.51								
After treatment	DWI	b1000	6.08 ± 1.71	–		–			–		
		b800	6.93 ± 2.57	1.432	0.158						
		b1600	4.64 ± 1.43	−3.399	0.001*						
	ADC Map	SS-EPI	162.59 ± 49.08	−7.108	< 0.001*						
		iShim	80.77 ± 35.44								

*P* with * suggested statistical significance. All the t and *P* values are the comparison between b = 800 s/mm^2^ of iShim-DWI and b = 1000 s/mm^2^ of SS-EPI-DWI, b = 1600 s/mm^2^ of iShim-DWI and b = 1000 s/mm^2^ of SS-EPI-DWI for each DWI item

**Fig. 2:**
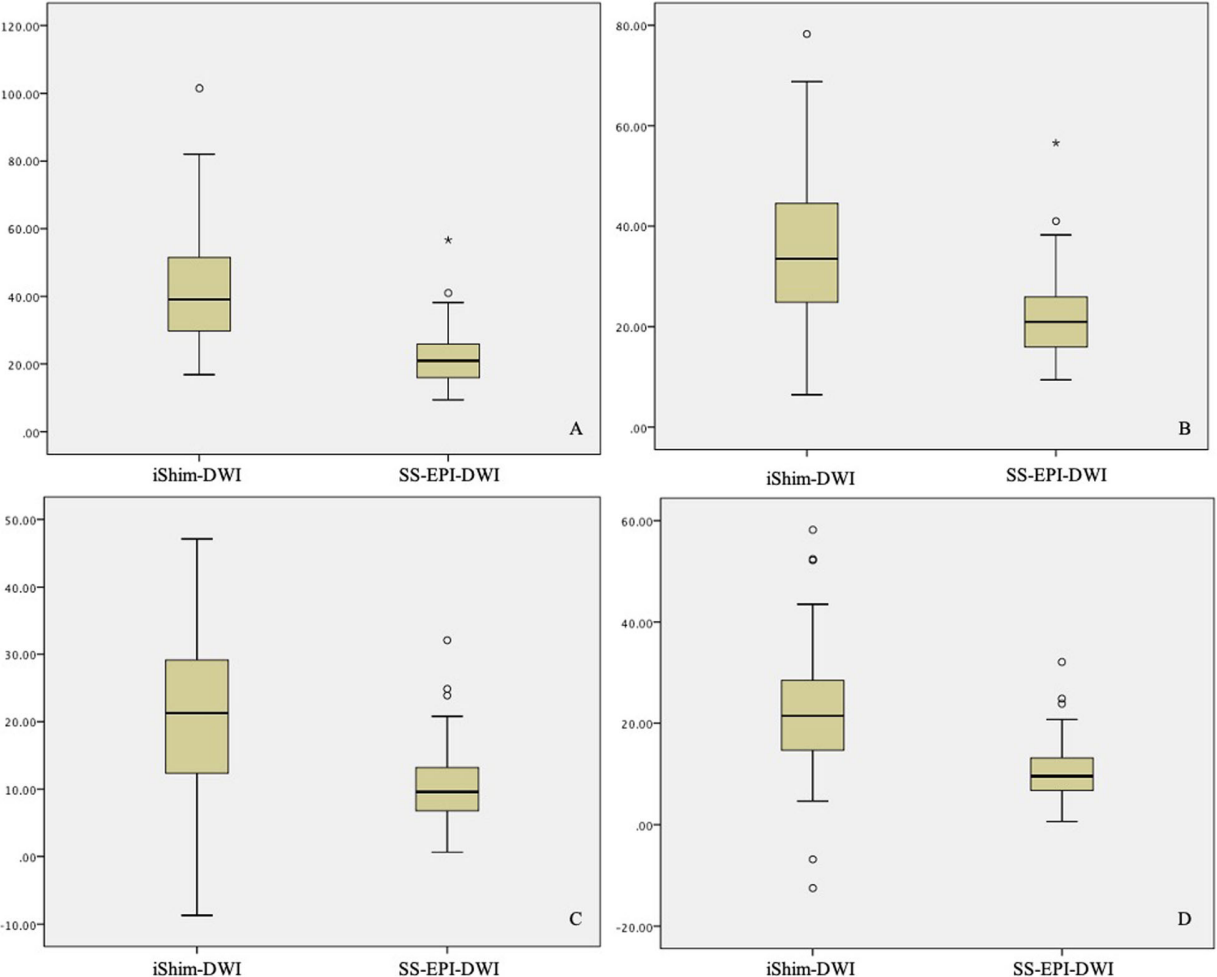
Boxplot of SNR and CNR of primary rectal tumor between iShim- and SS-EPI-DWI. **a**: SNR of primary rectal tumor in b = 800 s/mm^2^ of iShim-DWI was significantly higher than in b = 1000 s/mm^2^ of SS-EPI-DWI (*P* < 0.001). **b**: SNR of primary rectal tumor in b = 1600 s/mm^2^ of iShim-DWI was significantly higher than in b = 1000 s/mm^2^ of SS-EPI-DWI (*P* < 0.001). **c**: CNR of primary rectal tumor in b = 800 s/mm^2^ of iShim-DWI was significantly higher than in b = 1000 s/mm^2^ of SS-EPI-DWI (*P* < 0.001). **d**: CNR of primary rectal tumor in b = 1600 s/mm^2^ of iShim-DWI was significantly higher than in b = 1000 s/mm^2^ of SS-EPI-DWI (*P* < 0.001)

**Fig. 3:**
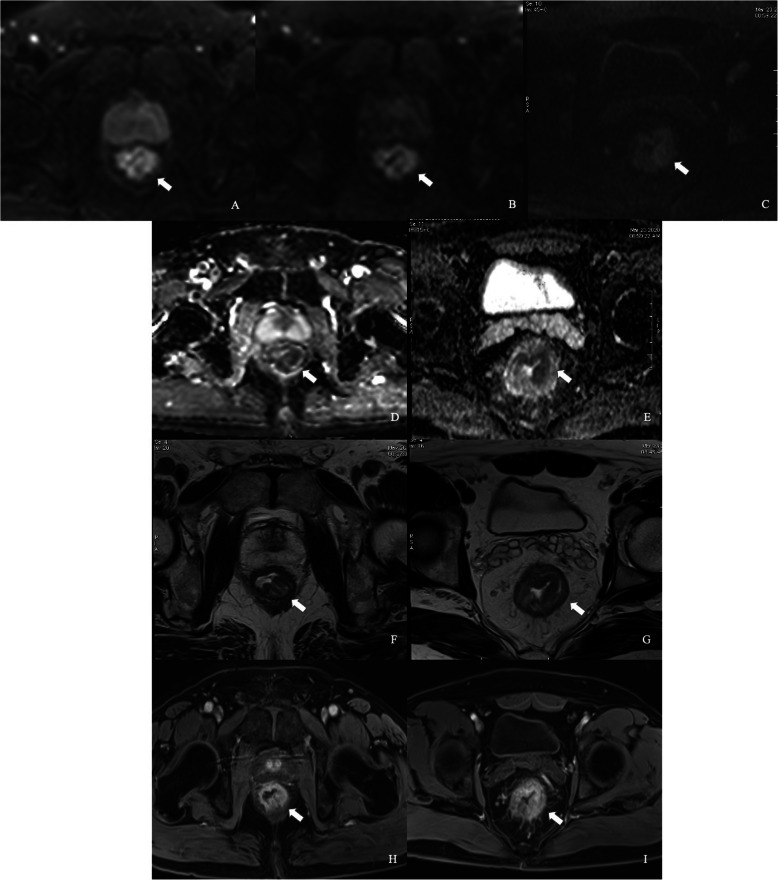
Comparison between iShim- and SS-EPI-DWI in patients with primary rectal cancer. iShim-DWI of b = 800 s/mm^2^ (**a**) and b = 1600 s/mm^2^ (**b**) showed higher SNR and CNR with lower signal noise compared with SS-EPI-DWI of b = 1000 s/mm^2^ (**c**). ADC map (**e**) of iShim-DWI showed better image quality compared with ADC map (**f**) of SS-EPI-DWI. T2W images (**f** and **g**) and dynamic T1W images (**h** and **i**) showed the same location of lesion (arrows) for iShim- and SS-EPI-DWI cohort, respectively

**Fig. 4:**
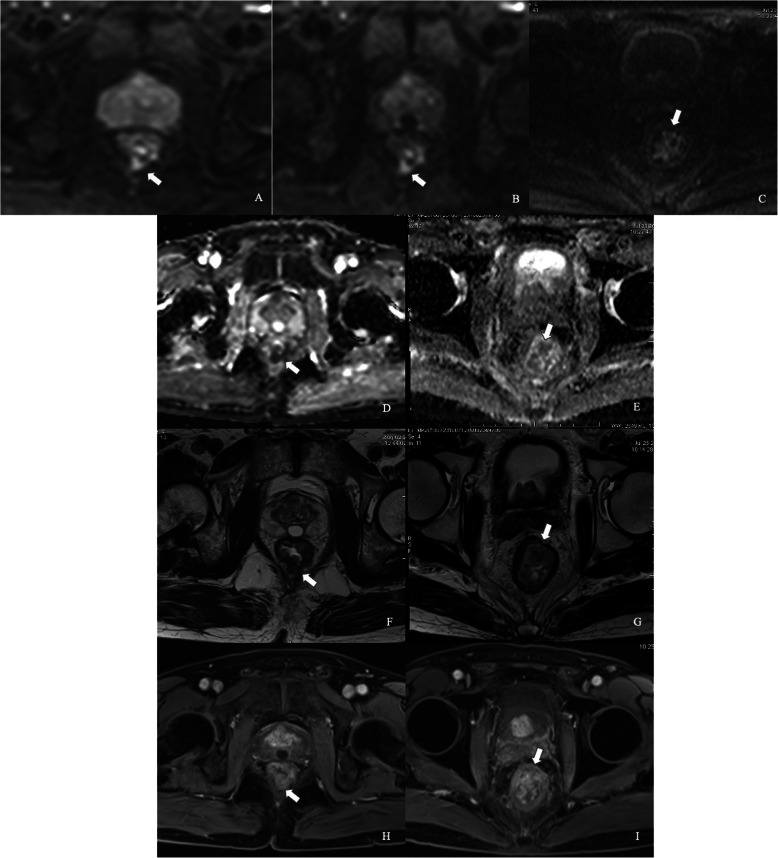
Comparison between iShim- and SS-EPI-DWI in patients after CRT. iShim-DWI of b = 800 s/mm^2^ (**a**) and b = 1600 s/mm^2^ (**b**) showed higher SNR and CNR with lower signal noise compared with SS-EPI-DWI of b = 1000 s/mm^2^ (**c**). ADC map (**e**) of iShim-DWI showed better image quality compared with ADC map (**f**) of SS-EPI-DWI. T2W images (**f** and **g**) and dynamic T1W images (**h** and **i**) showed the same location of lesion (arrows) for iShim- and SS-EPI-DWI cohort, respectively

Both SNR and CNR of b = 800 and 1600 s/mm^2^ images in iShim-DWI were significantly higher than those of b = 1000 s/mm^2^ images in SS-EPI-DWI. Meanwhile, b = 800 s/mm^2^ images showed the highest SNR among the 3 sets of DW images while b = 1600 s/mm^2^ images obtained the highest CNR (Table 2, Figs. 2, 3 and 4).

**Fig. 5:**
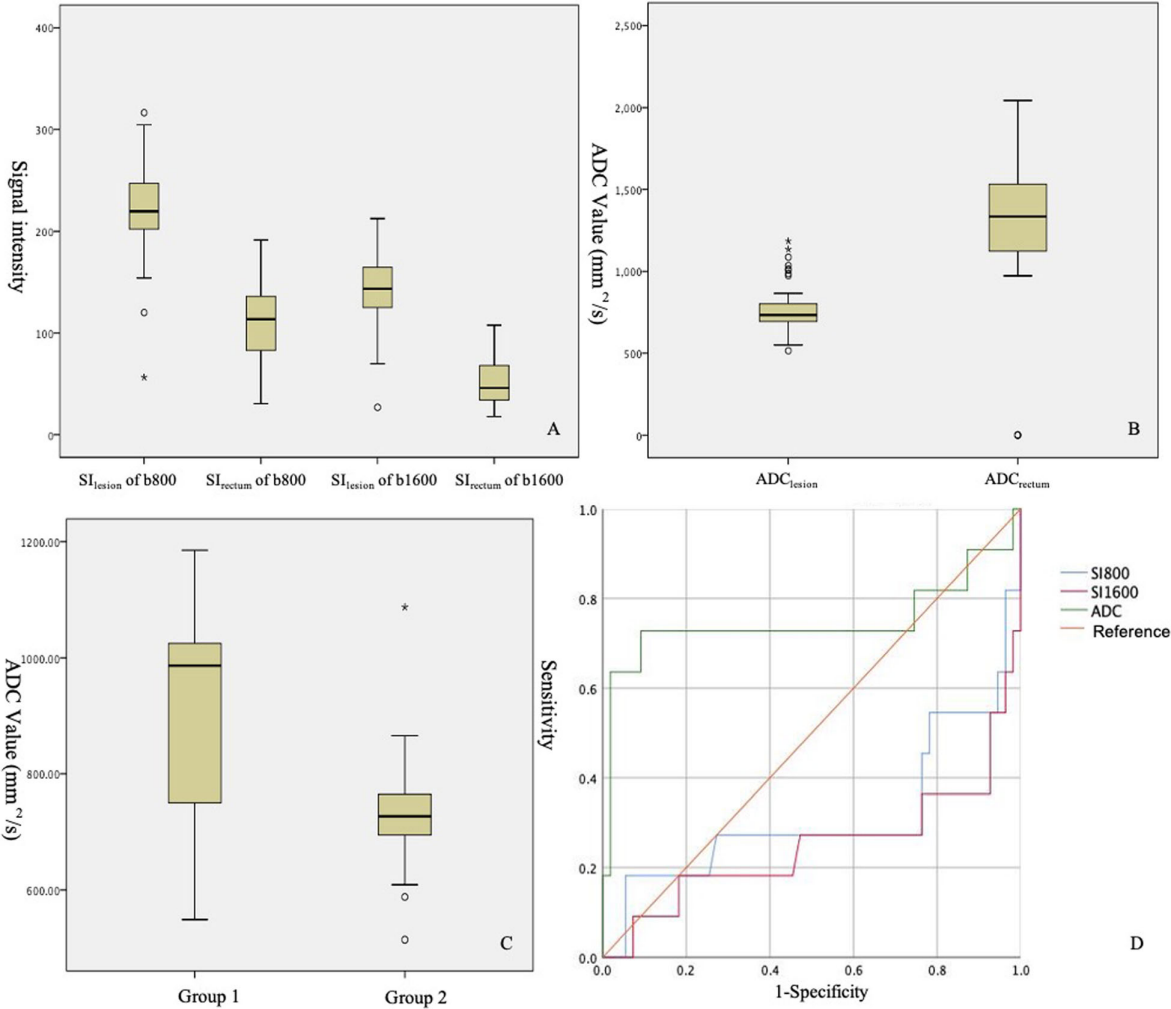
Boxplot of Quantitative Assessment of primary rectal cancer in iShim-DWI. **a**: Signal intensity (SI) showed significantly high in primary rectal cancer compared with residual rectal wall in iShim-DWI. **b**: ADC values shoed significantly low in primary rectal cancer compared with residual rectal wall in iShim-DWI. **c**: ADC values comparison showed significant difference between two pathological groups (Group 1: well differentiated cancer and Group 2: moderate to lower differentiated cancer). **d**: ROC analysis between two pathological groups showed statistical significance in SI_lesion_ of b = 800 s/mm^2^ (AUC = 0.692, *P* = 0.046), SI_lesion_ of b = 1600 s/mm^2^ (AUC = 0.756, *P* = 0.008) and ADC (AUC = 0.742, *P* = 0.012) maps, respectively

**Fig. 6:**
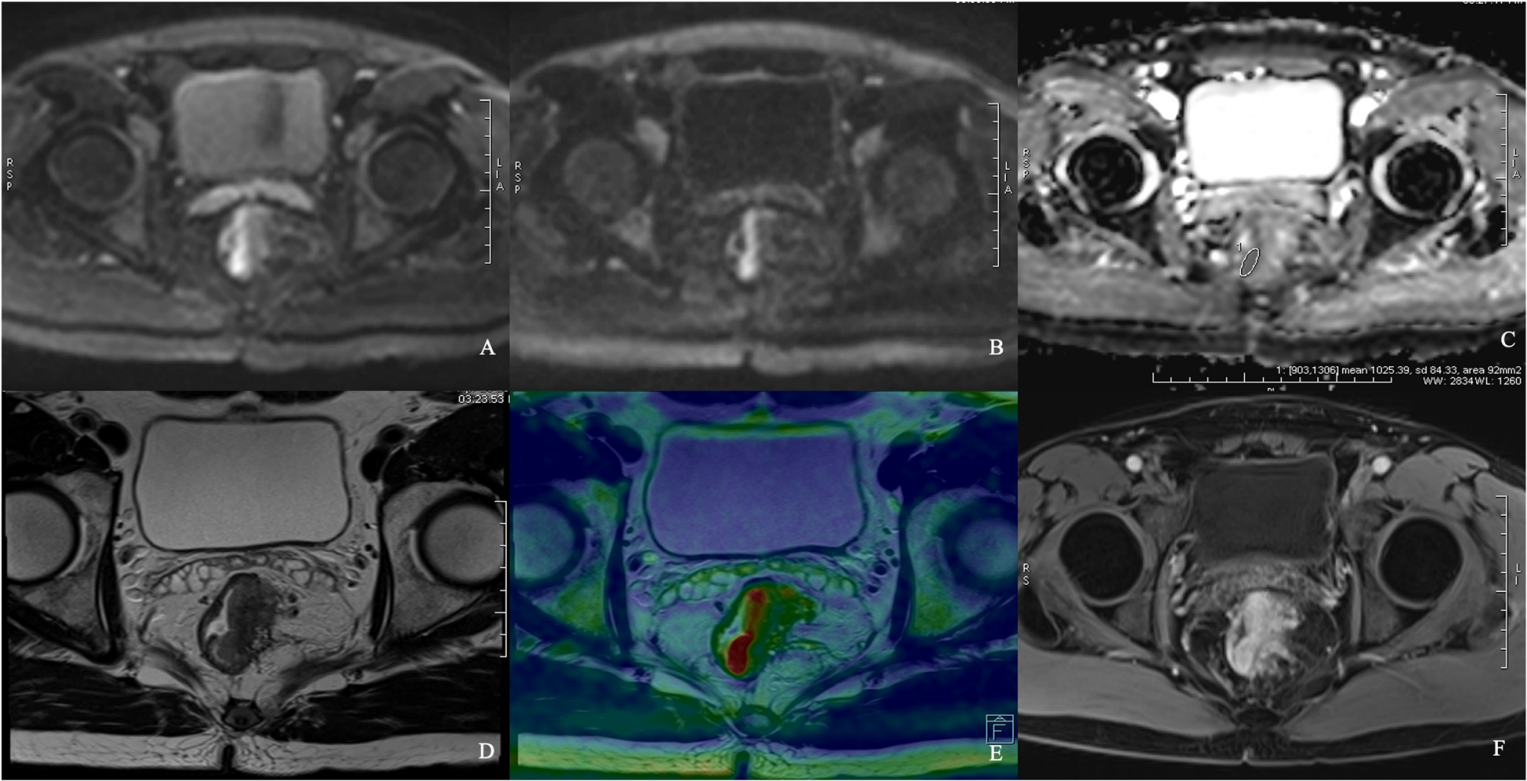
iShim DWI for patient with primary rectal cancer of Grade 1. DW images of b = 800 s/mm^2^ (**a**), b = 1600 s/mm^2^ (**b**), ADC map (**c**), T2WI (**d**), infusion images of both T2WI and DWI (**e**) and dynamic T1WI (**f**) showed the same location of lesion. Average ADC value measured was 1025.39 × 10^− 6^ mm^2^/s (**c**)

**Fig. 7:**
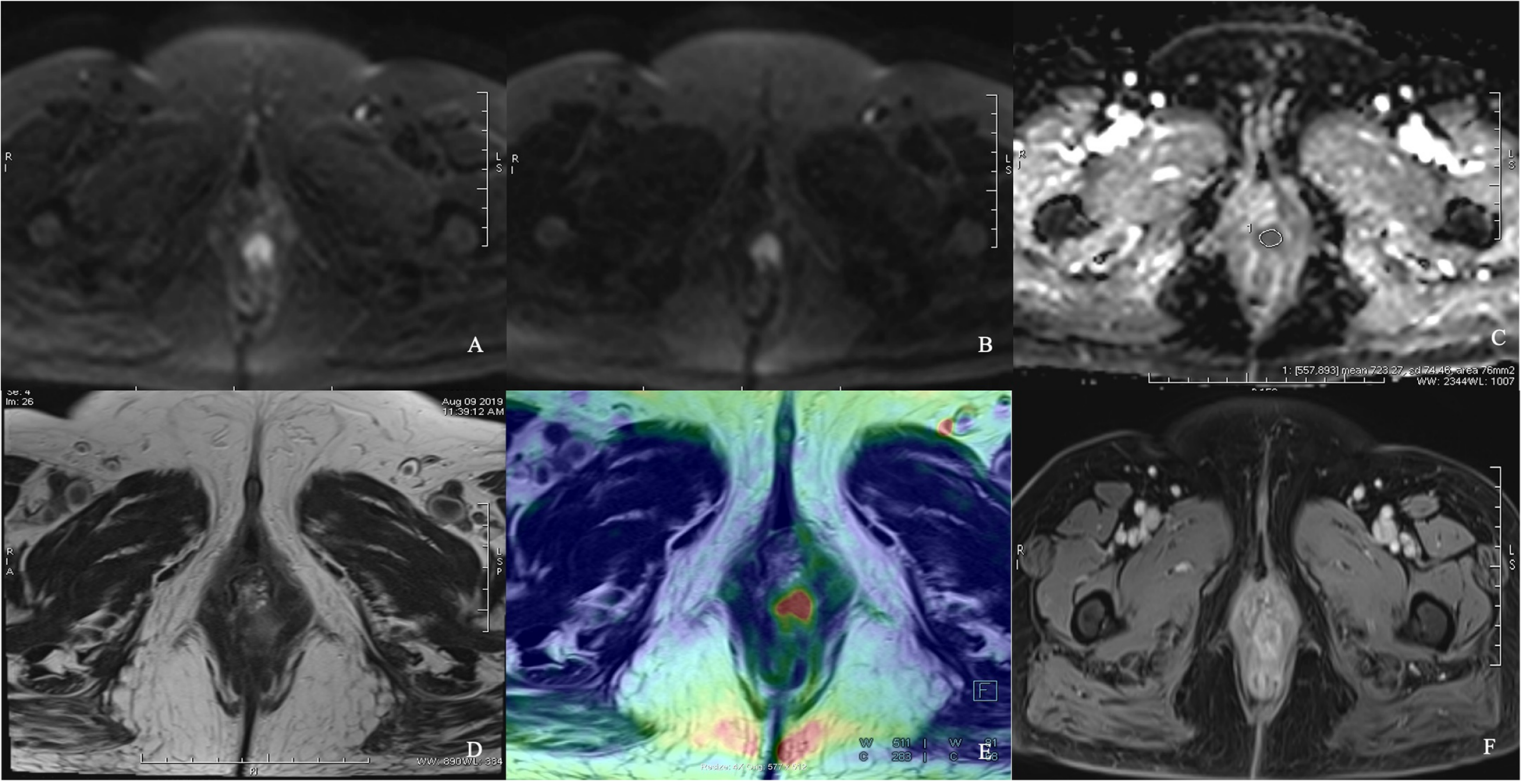
IShim DWI for patient with primary rectal cancer of Grade 3. DW images of b = 800 s/mm^2^ (**a**), b = 1600 s/mm^2^ (**b**), ADC map (**c**), T2WI (**d**), infusion images of both T2WI and DWI (**e**) and dynamic T1WI (F) showed the same location of lesion. Average ADC value measured was 723.27 × 10^− 6^ mm^2^/s (**c**)

-In 66 patients of primary rectal cancer of iShim-DWI, paired-*t*-test showed that SI_lesion_ was significantly higher than SI_rectum_ in both b = 800 and 1600 s/mm^2^ images. In addition, ADC_lesion_ was also significantly lower than ADC_rectum_. Between the two pathologic groups, ADC values was significantly lower in Group 2 (mean value: (0.733 ± 0.08) × 10^− 3^ mm^2^/s) than in Group 1 (mean value: (0.912 ± 0.21) × 10^− 3^ mm^2^/s). There was no significant difference between SI_lesion_ of b = 800 s/mm^2^ images, but SI_lesion_ of b = 1600 s/mm^2^ images showed significantly different between the two groups. ROC analyses showed significance of ADC values and SI_lesion_ between the two groups (Table 3, Figs. 5, 6 and 7).

**Table 3:** Results of Quantitative Assessment of primary rectal cancer

			*t* test			ROC analysis	
	Images		Average	t	*P*	Area under curve	*P*
SI_lesion_ SI_rectum_	b800	SI_lesion_	221.68 ± 43.51	16.868	< 0.001*	–	
		SI_rectum_	103.99 ± 42.68				
	b1600	SI_lesion_	143.28 ± 32.9	20.468	< 0.001*		
		SI_rectum_	48.94 ± 23.69				
	b1000	SI_lesion_	125.86 ± 32.14	17.441	< 0.001*		
		SI_rectum_	65.51 ± 15.57				
ADC_lesion_ ADC_rectum_	iShim-DWI	ADC_lesion_	0.763 ± 0.13	−11.103	< 0.001*		
		ADC_rectum_	1.294 ± 0.36				
	SS-EPI-DWI	ADC_lesion_	0.768 ± 0.13	−17.303	< 0.001*		
		ADC_rectum_	1.243 ± 0.22				
Group Comparison In iShim-DWI	ADC_lesion_ (×10^−3^ mm^2^/s)	Group 1	0.912 ± 0.21	2.837	0.017*	0.742	0.012*
		Group 2	0.732 ± 0.084				
	SI_lesion_ of b800	Group 1	191.91 ± 68.08	−1.697	0.118	0.692	0.046*
		Group 2	227.64 ± 34.71				
	SI_lesion_ of b1600	Group 1	113.95 ± 47.67	−2.379	0.036*	0.756	0.008*
		Group 2	149.15 ± 25.92				
Group Comparison In SS-EPI-DWI	ADC_lesion_ ((×10^−3^ mm^2^/s)	Group 1	0.815 ± 0.13	1.029	0.308	0.619	0.382
		Group 2	0.764 ± 0.1				
	SI_lesion_ of b = 1000	Group 1	125.1 ± 29.69	0.663	0.51	0.55	0.714
		Group 2	134.6 ± 40.71				

*P* with * suggested statistical significance

-In 60 patients of primary rectal cancer of SS-EPI-DWI, paired-*t*-test showed that SI_lesion_ was significantly higher than SI_rectum_ in b = 1000 s/mm^2^ images. In addition, ADC_lesion_ was also significantly lower than ADC_rectum_ (Table 3). In two pathologic groups of SS-EPI-DWI, there was no significant difference between them in both SI of DWI and in ADC values. ROC analyses also showed no significance in both SI_lesion_ and ADC values between the two groups (Table 3).

## Discussion

In this study, we investigated the qualitative and quantitative performance of iShim-DWI in rectal cancer imaging, compared with SS-EPI-DWI. The results demonstrated that iShim-DWI significantly outperformed SS-EPI-DWI in SNR and CNR of primary rectal tumor. Both SI and ADC values from iShim-DWI showed significant difference between primary rectal tumor and residual normal rectum. In addition, SI from b = 1600 s/mm^2^ images and ADC values have potential value in characterize well-differentiated and poor-differentiated rectal cancer.

DW image quality plays an important role in detection and characterization of lesions. Previous studies [16–22] have conducted research on improving image quality of DWI techniques such as iShim, readout segmentation of long variable echo trains (RESOLVE), multiplexed sensitivity-encoding (MUSE). Owing to the procedure of dynamic frequency adjustment and slice-selective shimming that reduces field inhomogeneities and thus related artifacts [18–20, 23], iShim-DWI showed superior performance in lesion detection of bladder, breast, neck and so on. Our study also demonstrated it could improve image quality in rectal tumor compared to SS-EPI-DWI and lesion detection with higher SNR and CNR. With the acceptable scanning time (less than 3 min), iShim-DWI has great potential application in routine work.

A few studies explored DWI with an ultra-high b-value (more than 1000 s/mm^2^) and showed it might be a promising technique for detecting malignant tumors [9–11]. For example, Bittencourt et al. found that DWI with b-value of 1400 s/mm^2^ showed benefits in increasing rates of detection of prostate cancer [9]. Also in our previous study of rectal cancer [10], we indicated that b-value of 2000 s/mm^2^ could be helpful in detecting lesions with a clear demarcation of borders. DWI with ultra-high b value could be helpful to obtain a nearly perfect background suppression (less ‘T2-shine-through’), but the image quality of b = 2000 s/mm^2^ did not showed any superiority when compared with that of b = 800 s/mm^2^, and even achieved lower scores in patients after CRT. However, DWI of b = 1600 s/mm^2^ achieved best image qualities and highest CNR compared to those of b = 800 and 1000 s/mm^2^ in both patients with primary rectal tumor and after treatment. Therefore, DWI of b = 1600 s/mm^2^ might be a better choice for ultra-high b value in rectal cancer in future study.

Another important reason for this result might be attributed to different DWI techniques used: iShim used in our study and SS-EPI in previous studies [9–11]. The surrounding tissue around rectum may induce inhomogeneity of DWI signal, such as bones, bowel movement and bowel contents. The advantage of iShim-DWI was to reduce the inhomogeneity and improve image quality. From our results, image quality showed higher in b = 800 s/mm^2^ of iShim-DWI than b = 1000 s/mm^2^ of SS-EPI-DWI. We speculated that iShim-DWI itself worth application in rectal tumor and will improve the lesion detection greatly.

ADC value, which is derived from DWI, is an important quantitative biomarker revealing lesion characterization. Previous studies have already investigated the use of SS-EPI-DWI as a tool for treatment response prediction and have shown that low pretreatment ADC values may correlate with a good response to CRT [24–27]. Another study also showed that an early increase of mean ADC value in rectal cancer correlates with a good response to CRT [28]. However, most studies focused on the investigation of ADC as a biomarker to assess or predict response to treatment. Few information of ADC on differentiation of primary rectal cancer has been known. And our resuts showed iShim-DWI may have the potential ability. As a non-invasive technique, this ability of iShim-DWI should gained more attention to for predicting pathological results.

However, in our study, ADC value generated by iShim-DWI combination of b = 0, 800 and 1600 s/mm^2^ could be helpful in differentiating rectal cancer with well- from poor-differentiation, but SS-EPI-DWI could not. From results of previous experiment, ADC value could be generated differently due to different b values combination [11]. Although iShim-DWI have improved the image quality with less artifacts, the technique might not be the main contributor to tumor differentiation. The combination of proper high and ultra-high b values should be the reason. More patients with well-differentiated rectal cancer should be enrolled in furture, and also we will continue to explore its treatment prediction in this cohort of patients with follow-ups.

Paired-*t*-test analysis showed SI of primary rectal cancer (in both b = 800 and 1600 s/mm^2^ DWI) significantly different from residual normal rectal wall in our study. The benefits for this result might help determine the borderline of tumor more precisely. However, an overlap existed in both SI and ADC value between the two tissue. Moreover, the normal rectal wall was always too thin to be drawn ROI on. Small field of view (FOV) techniques combined with iShim-DWI might help further differentiation in future.

There are several limations in our study. First, our study may have statitical bias, because patients enrolled were received different DWI protocols. Considering the heavy clinical workload in routine work, repeat scanning for every patient will increase the amont of the work. However, before the study, we conducted both SS-EPI-DWI and iShim-DWI in 2 patients for feasibility experiment, and our results showed the image quality were higher in both DWI images and ADC maps of iShim-DWI compared with SS-EPI-DWI. Consecutive patients were selected with DWI protocols randomly applied. In addition, large sample capacity (nearly 100 patients for each cohort) enrolled in our study also could reduce the bias and make our results persuasive. Second, lesion measurement was conducted on 2-dimentional images which may not be as accurate as measuring the whole tumor in three dimensions. To comprensate for that, we used multiple sites measurement to avoid bias as much as possible. In the future, volumetric measurement could be explored by development of measurement tools. Another limitation was the relatively small capacity of well-differentiated cancer enrolled. Following study should be further conducted with more participants enrolled.

## Conclusion

In conclusion, iShim-DWI with b values of 800 and 1600 s/mm^2^ provides improved spatial resolution and image quality for rectal cancer imaging and characterization.

## Supplementary Information

**Additional file 1.**

## Data Availability

Not applicable.

## Abbreviations

DWI: Diffusion-weighted imaging
SS-EPI: Single-shot echo-planar imaging
SI: Signal intensity
CRT: Chemo-radiation therapy
ADC: Apparent diffusion coefficient
iShim: Integrated slice-specific dynamic shimming
T2W TSE: T2-weighted Turbo Spin Echo
PACS: Picture archiving and communication systems
ROI: Region of interest
SD: Standard deviation
SNR: Signal-noise ratio
CNR: Contrast-to-noise ratio
ROC: Receiver operator curve
RESOLVE: Readout segmentation of long variable echo trains
MUSE: Multiplexed sensitivity-encoding
FOV: Field of view
