# Supplementary material for: Integrated slice-specific dynamic shimming diffusion weighted imaging (DWI) for rectal Cancer detection and characterization
Source: Cancer Imaging. 2021 Apr 7;21:32. doi: 10.1186/s40644-021-00403-9 (PMC8028796; doi:10.1186/s40644-021-00403-9)
Supplement: Supplementary file 1 — Additional file 1. [file 40644_2021_403_MOESM1_ESM.docx]

**Supplement 1 DWI parameters for rectal MR imaging**

| Parameters | T2_TSE | T2_TSE | T2_TSE_FS | ss-EPI  DWI | iShim  DWI | DCE  T1_VIBE_Dixon |
| --- | --- | --- | --- | --- | --- | --- |
| Scan Plane | Axial | Sagittal | Coronal | Axial | Axial | Axial |
| TR/TE | 7000/93 | 3000/97 | 6400/84 | 5700/63 | 6300/60 | 6.69/2.39, 4.77 |
| Matrix | 320*224 | 384*288 | 320*240 | 128*128 | 128*128 | 320*240 |
| Slices | 30 | 20 | 20 | 30 | 24 | 48 |
| Thickness (mm) | 3 | 5 | 3.5 | 3.5 | 4.5 | 3 |
| B value (s/mm^2^) |  |  |  | 50,1000 | 0,800,  1600 |  |
| Averages |  |  |  | 4 | 12 |  |
| iPAT | 2 | 2 | 0 | 2 | 2 | 2 |
| Fat Suppression |  |  | SPAIR | SPAIR | SPAIR | Dixon |
| Bandwidth |  |  |  | 1446 | 2442 |  |
| Scan Time, (min:sec) | 3:16 | 1:54 | 2:21 | 2:23 | 2:56 | 3:43 |

Note: DWI =diffusion-weighted imaging, TSE, turbo spin echo; TR/TE, repetition time/echo time; FOV, field of view; FS, fat saturation; iShim­EPI, integrated­shimming echo planar imaging; SS-EPI: single-shot echo planar imaging; iPAT: integrated parallel acquisition techniques; SPAIR =spectral attenuation inversion recovery.
